# TRPA1 expression and its functional activation in rodent cortex

**DOI:** 10.1098/rsob.160314

**Published:** 2017-04-19

**Authors:** Ehsan Kheradpezhouh, Julian M. C. Choy, Vincent R. Daria, Ehsan Arabzadeh

**Affiliations:** 1 Eccles Institute of Neuroscience, John Curtin School of Medical Research, Australian National University Node, Acton, Australian Capital Territory 2601, Australia; 2 The Australian Research Council Centre of Excellence for Integrative Brain Research, Australian National University Node, Acton, Australian Capital Territory 2601, Australia

**Keywords:** cortex, trpa1, layer 5 pyramidal neurons, optovin, photoswitching

## Abstract

TRPA1 is a non-selective cation channel involved in pain sensation and neurogenic inflammation. Although TRPA1 is well established in a number of organs including the nervous system, its presence and function in the mammalian cortex remains unclear. Here, we demonstrate the expression of TRPA1 in rodent somatosensory cortex through immunostaining and investigate its functional activation by whole-cell electrophysiology, Ca^2+^ imaging and two-photon photoswitching. Application of TRPA1 agonist (AITC) and antagonist (HC-030031) produced significant modulation of activity in layer 5 (L5) pyramidal neurons in both rats and mice; AITC increased intracellular Ca^2+^ concentrations and depolarized neurons, and both effects were blocked by HC-030031. These modulations were absent in the TRPA1 knockout mice. Next, we used optovin, a reversible photoactive molecule, to activate TRPA1 in individual L5 neurons of rat cortex. Optical control of activity was established by applying a tightly focused femtosecond-pulsed laser to optovin-loaded neurons. Light application depolarized neurons (*n* = 17) with the maximal effect observed at *λ* = 720 nm. Involvement of TRPA1 was further confirmed by repeating the experiment in the presence of HC-030031, which diminished the light modulation. These results demonstrate the presence of TRPA1 in L5 pyramidal neurons and introduce a highly specific approach to further understand its functional significance.

## 1. Introduction

Transient receptor potential ankyrin 1 (TRPA1) is a member of the ancient superfamily of transient receptor potential (TRP) channels that are generally involved in pain, thermal and chemical sensation [1,2]. TRPA1 is a non-selective cation channel, exhibits high structural similarity to TRP vanilloid 1 (TRPV1), and contains the longest N-terminal among all known TRP channels in vertebrates [1,3,4]. The N-terminal contains 14–18 ankyrin repeat domains which represent the binding site for TRPA1 activators including non-specific irritants and Ca^2+^ [1,3–6]. TRPA1 is broadly expressed throughout the body including brain, heart, small intestine, lung, bladder, joints and skeletal muscles [7–15]. As a mechanosensor, TRPA1 is expressed in the peripheral sensory pathways and in the inner ear [16–18]. TRPA1 is prominently present in the nociceptive neurons of dorsal root ganglia (DRG) and the trigeminal ganglia [19], and is activated by a range of irritants including mustard oil, acrolein, formalin and 4-hydroxynonenal [1,3,20–22]. In the central nervous system, TRPA1 is studied in the hippocampus [14] and the supraoptic nucleus of the brain stem [7,23].

Despite the widespread agreement about the role of TRPA1 in pain sensation and neurogenic inflammation, our knowledge of its expression and function in the cerebral cortex remains minimal. TRPA1 loses its functionality in excised membrane patches due to several biophysical and cellular regulatory sites [4,24,25] which have been confirmed by the recent discovery of TRPA1's molecular structure [26]. Non-specific agonists have been used to study TRPA1 function including pungent molecules extracted from wasabi, garlic and cinnamon [20,27–29] and synthetic compounds such as unsaturated aldehydes, trinitrophenol and 2-aminoethoxydiphenyl-borate (2-APB) [4,24,25]. Although known TRPA1 agonists often activate other TRP channels (but see [30]), there are several specific TRPA1 antagonists including AP-18 [31], Chembridge-5861528 [32,33], A-967079 [34] and HC-030031 [35]. Recently, optovin—a small synthetic molecule—was used to specifically target TRPA1 channels and to produce motor behaviours in wild-type zebrafish [36]. Optovin contains an α,β-unsaturated rhodanine system, an olefin-like compound that reacts with the cysteine residue of TRPA1. Besides its TRPA1-binding site, optovin contains a rhodopsin-photoactive site; application of violet light (387 nm) reversibly photoswitches optovin which in turn activates TRPA1 channels [36]. The optovin photoswitching thus allows activation of TRPA1 at high spatial and temporal precision in order to study its function in brain tissue.

Here, we first demonstrate the presence of TRPA1 in rodent somatosensory cortex with immunostaining, calcium imaging and whole-cell recording. We then use optovin photoswitching to probe TRPA1 function in cortical neurons at high temporal resolution. Rather than single-photon photoswitching with violet light, we achieve a highly localized excitation method via nonlinear two-photon photoswitching. Optovin photoswitching thus allows us to specifically gate TRPA1 channels and to activate individual layer 5 (L5) pyramidal neurons. Two-photon excitation uses a near-infrared (NIR) femtosecond (fs) pulsed laser, which penetrates deeper into the brain tissue.

## 2. Material and methods

### 2.1. Preparation of brain slices

Coronal brain slices (300 µm) were prepared from 28–35 day old male Wistar rats, wild-type C57Bl/6 J mice and TRPA1 knockout (TRPA1-KO) mice; the brain was sliced with a vibratome (DSK Microslicer^®^) in ice-cold oxygenated artificial cerebrospinal fluid (aCSF) containing (in mM): 1.25 NaH_2_PO_4_, 1.0 MgCl_2_, 125.0 NaCl, 2.5 KCl, 2.0 CaCl_2_, 25.0 NaHCO_3_ and 10.0 glucose. Slices were then incubated at 34°C for 30 min in a holding chamber containing carbogen (5% CO_2_/95% O_2_) bubbled aCSF and then kept at room temperature in the same chamber.

### 2.2. Two-photon Ca^2+^ imaging

Neurons were loaded with the Ca^2+^ indicator by incubating the slices in carbogen-bubbled aCSF containing 5 µM Cal-520 acetoxymethyl ester (Cal-520 AM; AAT Bioquest, Sunnyvale, CA, USA) at room temperature for 45 min. After three episodes of 5 min washing with carbogen-bubbled aCSF, the slices were transferred to the chamber for fluorescence imaging. Slices were continuously perfused with 36 ± 1°C carbogen-bubbled aCSF containing (in mM): 1.25 NaH_2_PO_4_, 1.0 MgCl_2_, 125.0 NaCl, 2.5 KCl, 2.0 CaCl_2_, 25.0 NaHCO_3_ and 25 glucose at the rate of 2–3 ml min^−1^. Ca^2+^ imaging was performed using a two-photon microscope (Thorlabs Inc., Newton, NJ, USA) controlled by ThorImage OCT software. The brain slice was illuminated with a Ti:Sapphire fs-pulsed laser (Chameleon, Coherent Inc., Santa Clara, CA, USA) tuned at 810 nm. The laser was focused through a 16× water-immersion objective lens (0.8NA, Nikon) onto the tissue and Ca^2+^ transients were obtained from neuronal populations at a resolution of 512 × 512 pixels (sampling rate, 10 Hz). The change in fluorescence activity (Δ*F*/*F*) was quantified after importing images into ImageJ software (National Institutes of Health, USA) and by manual selection of the regions of interest (ROIs) around the neuronal cell bodies.

### 2.3. Whole-cell electrophysiology

After transferring the slices to the recording chamber, visualized whole-cell patch-clamp recording was performed on L5 pyramidal neurons in the somatosensory cortex using differential interference contrast (DIC) video microscopy. Slices were continuously perfused with 36 ± 1°C carbogen-bubbled aCSF containing (in mM): 1.25 NaH_2_PO_4_, 1.0 MgCl_2_, 125.0 NaCl, 2.5 KCl, 2.0 CaCl_2_, 25.0 NaHCO_3_ and 25 glucose at the rate of 2–3 ml min^−1^. The neuronal viability and functionality were tested by recording the membrane's voltage and action potentials in response to a depolarizing current pulse in the current clamp mode. The internal solution contained (in mM): K-gluconate, 115; KCl, 20; HEPES, 10; phosphocreatine, 10; Mg-ATP, 4; Na-GTP, 0.3 and biocytin, 0.25%. The pH of the internal solution was titrated with 1M KOH to a final pH of 7.3 and the osmolarity adjusted to 305 mO sml^−1^ with water or sucrose. The recording pipette contained 0.25% biocytin (Sigma-Aldrich Co. LLC., Rockville, MD, USA) for morphological reconstruction and optovin (Tocris Bioscience, Bristol, UK). Recording of the membrane currents was made in whole-cell voltage clamp mode using a MultiClamp 700B amplifier (Molecular Devices, Sunnyvale, CA, USA). Bridge balance and capacitance neutralization were carefully adjusted and checked for stability. A series of current steps (duration 500 ms, amplitudes ranging from −80 to 500–2000 pA in increments of 40 pA) was applied to identify neuronal firing patterns. Action potentials were recorded in whole-cell current clamp mode using the same amplifier. Only cells with a stable resting membrane potential from the start of the patch were chosen for recording. Voltage or current traces were filtered at 10 kHz and digitized at 20–50 kHz by an ITC-18 interface (HEKA Instruments Inc., Holliston, MA, USA) under the control of Axograph software (Axograph Scientific, Berkeley, CA, USA). To modulate TRPA1 activity, we applied AITC (TRPA1 agonist, 1 mM) and HC-030031 (TRPA1 antagonist, 10 µM) through the bath solution. Although lower concentrations of AITC were effective in activation of TRPA1 in transfected HEK cells [35], we selected a high concentration (1 mM) based on previous experiments on native cells [11,37–39]. Similarly we used a concentration of 10 µM for HC-030031, compatible with previous studies [35].

### 2.4. Two-photon activation of optovin-loaded neurons

Pyramidal neurons were filled with 0.1 mM Alexa-488 and imaged using a custom-built two-photon microscope [39] that allowed for visualizing the sample via an upright DIC image (Olympus BX50WI) to facilitate patching of neurons in 300 µm thick brain slices. In DIC imaging mode, the dichroic mirror (DM) above the objective lens allowed NIR light (*λ* > 900 nm) to pass through and focus onto a charge-coupled device (CCD) camera (Dage-MTI IR-1000EX). In the two-photon mode, a set of xy galvanometer (GM) scanning mirrors scanned the incident laser for raster imaging and for random positioning of focal stimulation of the neurons. The microscope used a 40× objective lens (Zeiss 1.0NA). Custom software developed in Labview (National Instruments) controlled the acquisition of three-dimensional (3D) images, and the laser intensities via a polarizing beam splitter and a half-wave plate on a motorized rotation mount. During optovin activation, the change in membrane potential was monitored in whole-cell current clamp. Two separate lasers were used, one for imaging (Coherent Mira 900 pumped with 12 W Verdi G) and one for two-photon photoswitching of optovin (Coherent Chameleon, wavelength tuneable from *λ* = 700 to 1100 nm). Two-photon imaging was acquired by setting the laser at *λ* = 850 nm and 5–10 mW average power. For two-photon photoswitching, the laser was set to *λ* = 700 to 800 nm at 10–20 mW average power. After breaking into the cell, we waited for a minimum of 3 min to allow optovin perfusion, before applying the laser. TRPA1 activation was limited to the recorded neuron because the excitation was localized within the diameter of the laser focus (approx. 1 µm) [40]. We activated TRPA1 along the dendritic shaft close to the soma. To identify the optimal stimulation parameters, we applied the following six wavelengths: *λ* = 700, 720, 730, 760, 780 and 800 nm. Every trial contained a 100-ms light stimulation with an inter-trial interval of 1300 ms. Different wavelengths and intensities were applied in blocks of 20 trials. To allow recovery from stimulation, and for the membrane potential to return to baseline, we applied a 1-min pause between consecutive blocks. These blocks were interleaved and repeated in a pseudorandom order to produce a total of 60 trials per each wavelength/intensity combination.

### 2.5. Immunohistochemistry

At the completion of the experiment, the brain slices were fixed in 4% paraformaldehyde in phosphate buffered saline (PBS) at 4°C. After fixation, the slices were rehydrated gradually by stepwise incubation in 10 to 30% sucrose in PBS (w/v). After rehydration, the slides were sectioned with a Leica CM1580 cryostat at 60 µm thickness and penetrated using PBS containing 1% Triton-X (v/v) for 4–5 h at room temperature under continuous shaking. The slides were washed three times with PBS at room temperature. To visualize the biocytin loaded neurons, the slices were incubated with streptavidin Alexa Fluor® 488 conjugate (Thermo Fisher Scientific, Waltham, MA, USA) overnight at 4°C on a shaker. To investigate the TRPA1 expression, the slices were incubated first with rabbit anti-TRPA1 primary antibody (AB58844, Abcam, Cambridge, UK) overnight at 4°C on a shaker, washed three times, and then incubated with either goat (488) or donkey (555) anti-rabbit IgG Alexa Fluor^®^ secondary antibody (Abcam, Cambridge, UK). To stain the nuclei of cortical cells, the slices were briefly (approx. 10 min) incubated with 4′,6-diamidine-2′-phenylindole dihydrochloride (DAPI) (Sigma-Aldrich Co. LLC., Rockville, MD, USA) at room temperature on a shaker. After washing, the slices were mounted on glass slides using PDX New (Merck, Darmstadt, Germany) and dried. Images were captured with a Nikon A1 confocal microscope. Laser-generated excitation wavelengths of 405, 488 and 555 nm were used to detect DAPI (nuclei), Alexa Fluor^®^ 488 (biocytin and TRPA1) and Alexa Fluor^®^ 555 (TRPA1), respectively. The images were then processed by ImageJ (JACoP Plugin) and colocalization scatter plots were created in MATLAB (Mathworks, Natick, MA, USA).

## 3. Results

### 3.1. Cortical expression of TRPA1

**Figure 1.**
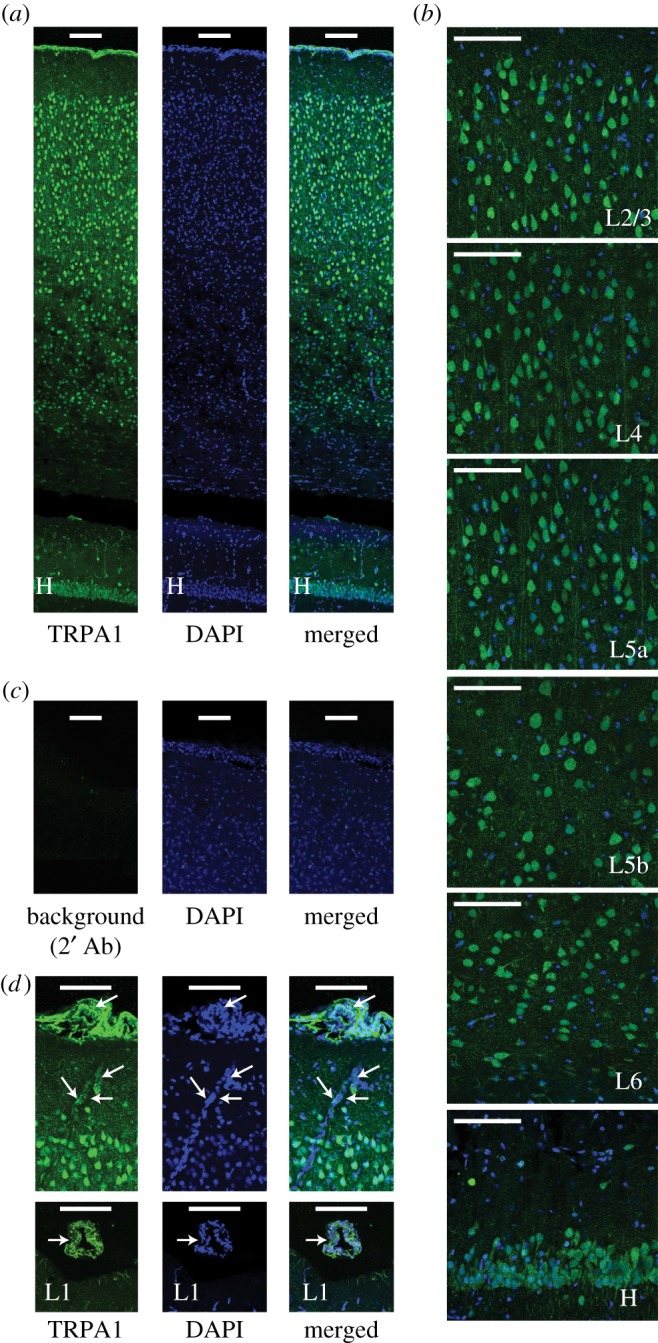
TRPA1 expression in the rat cortex. (*a*) Confocal images of a cortical slice (coronal section) capture anti-TRPA1 antibody (green) merged with nuclear staining with DAPI (blue). (*b*) TRPA1 expression in neurons across all cortical layers (L2/3, L4, L5a, L5b and L6) and in the hippocampus (H). (*c*) Incubation of the brain sections with secondary antibody alone (2′ Ab) shows minimal non-specific binding of the secondary antibody to the brain slices. (*d*) TRPA1 is prominently expressed in cortical blood vessels (arrows). Scale bar, 100 µm in all panels.

In the first step, we investigated the expression of TRPA1 in the rat somatosensory cortex. We prepared coronal brain slices from male Wistar rats aged 28–35 days and determined TRPA1 expression by application of TRPA1-specific antibodies (figure 1*a*). We discovered TRPA1 expression in neurons across all cortical layers (figure 1*b*). Beyond cortical neurons, the immunostaining results also revealed expression of TRPA1 in hippocampal neurons (H in figure 1*b*) and cortical vessels (figure 1*d*) as previously reported [7,26,41]. The level of TRPA1 expression in cortical neurons seems comparable with that of the hippocampal neurons.

**Figure 2.**
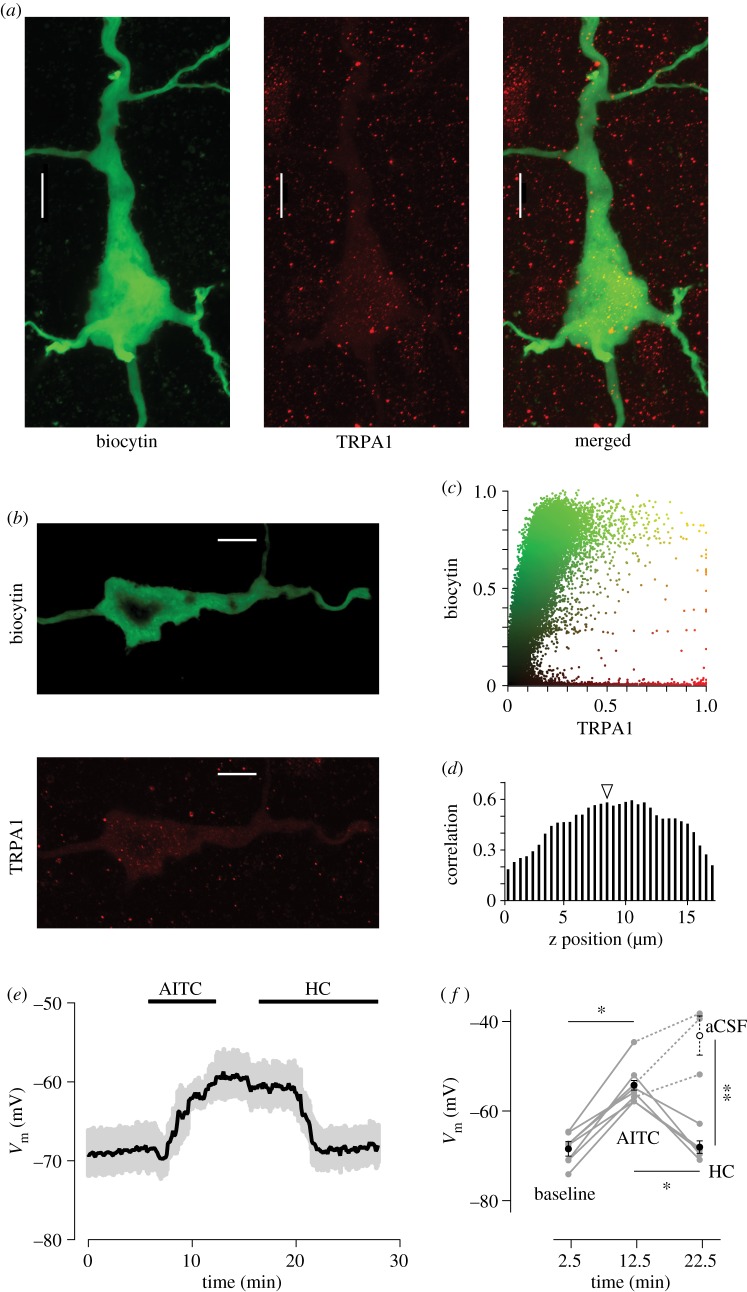
TRPA1 expression in individual L5 pyramidal neurons. (*a*) Z-stacked confocal images of an L5 pyramidal neuron loaded with biocytin (green) and visualized by tagged-streptavidin (left panel). TRPA1 expression reveals the outline of the neuron (middle panel). The merged image shows the overlap between biocytin and TRPA1 (right panel). A total of 33 z-stacks at 0.5-µm spacing were combined to generate each panel. Scale bar, 10 µm. (*b*) A single z-stack image of the neuron shown in (*a*). (*c*) The scatterplot shows the normalized fluorescent values at every pixel. Every dot is coloured by combining the relative fluorescent intensities in green (biocytin) and red (TRPA1) fluorochromes. The scales are normalized from 0–1 for green and red fluorochromes. (*d*) Pearson's correlation coefficient values (correlation) at each z position (0–17 µm) of the neuron shown in (*a*). Z-stack images were acquired through confocal microscopy with 0.1 µm thickness at 0.5 µm intervals. The arrow indicates the correlation value for the section shown in (*b*). (*e*) Whole-cell recording of L5 pyramidal neurons reveal depolarization with TRPA1 agonist AITC. The shaded area indicates s.e.m. of *V*_m_ across neurons (*n* = 5). By replacing AITC with specific TRPA1 antagonist HC-030031 (HC), the membrane potential returned to its baseline values (hyperpolarized). (*f*) *V*_m_ plotted individually for the eight neurons (grey circles) at each critical time point: at baseline (baseline, 2.5 min, *n* = 8); after application of agonist (AITC, 12.5 min, *n* = 8); after application of antagonist (HC, 22.5 min, *n* = 5); and after prolonged wash (aCSF, 22.5 min, *n* = 3). Black circles with s.e.m. show mean values at each time point. *p < 0.001, **p = 0.025.

To further examine the expression of TRPA1 in neurons, we loaded individual L5 pyramidal cells with biocytin using patch pipettes and quantified the colocalization of TRPA1 and biocytin using confocal microscopy (figure 2*a*,*b*). Across 156 non-overlapping z-stack images captured from three reconstructed neurons, we found that colocalization was in the range of 0.19–0.67 (Pearson's correlation coefficient) with a mean ± s.e.m. of 0.42 ± 0.13 (figure 2*c*,*d*). A mirror rotation of the biocytin (green) images significantly reduced the correlation values (paired *t*-test, *p* < 0.0001) to 0.02–0.09 at all z-stack images indicating that TRPA1 expression followed the outline of the neuron. A critical next step was to verify whether TRPA1 could be functionally activated in cortical neurons. We investigated this question with electrophysiological and Ca^2+^ imaging experiments conducted in the presence and absence of TRPA1 agonist and antagonist.

### 3.2. TRPA1 modulation of the membrane potential

To examine the physiological effect of TRPA1 activation on neurons, we performed *in vitro* whole-cell recording of L5 pyramidal cells (figure 2*a*) and measured the changes in membrane potential (*V*_m_) in the presence of TRPA1 agonist (allyl isothiocyanate, AITC) and TRPA1 antagonist (HC-030031). Application of 1 mM AITC increased *V*_m_ significantly (depolarization, *n* = 8, *p* < 0.0001). AITC is a well-recognized TRPA1 agonist gating the channel by reversible covalent binding to the N-terminal [42]; however, it is not selective to TRPA1 only and can exert its effect through non-specific binding to other TRP channels [30,43]. In order to see whether the effect observed in figure 2*e* was through activation of TRPA1 channels, in a subset of neurons (*n* = 5) we followed the application of AITC with 10 µM HC-030031, a highly selective TRPA1 antagonist. After a few minutes of exposure to HC-030031, *V*_m_ returned towards its baseline levels (*n* = 5, *p* < 0.0001, figure 2*f*). In another subset of neurons (*n* = 3), the application of AITC was followed by more than 20 min of washing with aCSF (figure 2*f*). This allowed us to discriminate between the antagonistic activity of HC-030031 and a non-specific wash-out of AITC. The return to baseline *V*_m_ was only observed in the HC-030031 group with no decline in *V*_m_ for the prolonged wash (paired *t*-test, *p* < 0.001).

Next, we asked if these changes in membrane potential also corresponded to changes in intracellular Ca^2+^. To examine this we employed two-photon Ca^2+^ imaging, which further allowed us to test the effect of TRPA1 activation on a population of neurons.

### 3.3. TRPA1 modulation of neuronal Ca^2+^

**Figure 3.**
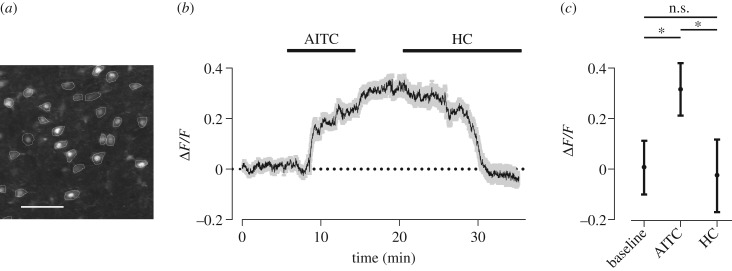
TRPA1 activation increases intracellular calcium. (*a*) The two-photon image shows 27 L5 cells loaded with Cal-520 in one imaging session. The regions of interest were manually selected at the margin of the cells. Scale bar, 50 μm. (*b*) Five minutes after the onset of imaging, AITC was applied to the bath, followed by washing and then by application of HC-030031 (HC). The grey shaded area indicates s.e.m. of Δ*F*/*F* across neurons (*n* = 79) recorded in three imaging experiments. (*c*) Δ*F*/*F* measured at three time points after the onset of imaging: 2.5 min (baseline), 12.5 min (AITC) and 32.5 min (HC). Δ*F*/*F* is measured over a 1 s duration for each cell and averaged across cells (*n* = 79). Error bars indicate s.d. of Δ*F*/*F* across neurons. *p < 0.001, n.s., *p* > 0.05.

We conducted two-photon Ca^2+^ imaging on brain slices prepared from 28–35-day-old Wistar rats and measured the relative changes in the cytosolic Ca^2+^ concentration ([Ca^2+^]_c_) of L5 pyramidal neurons. As TRPA1 channels are more permeable to Ca^2+^ than other cations [44], activation of TRPA1 by agonists is expected to produce a measurable increase in neuronal [Ca^2+^]_c_. We quantified the relative changes in fluorescence (Δ*F*/*F*) after application of AITC and HC-030031. Introducing AITC in the bath solution significantly increased Δ*F*/*F* indicating a rise in neuronal [Ca^2+^]_c_ (paired *t*-test, *p* < 0.001). After replacing AITC with 10 µM HC-030031, the neuronal [Ca^2+^]_c_ returned to its baseline values (figure 3).

### 3.4. Modulations are absent in the TRPA1-KO mice

In earlier results, whole-cell electrophysiology and Ca^2+^ imaging revealed significant modulation of activity in L5 pyramidal neurons by TRPA1 agonist and antagonist. A powerful method to verify that the observed functional modulations were indeed through the TRPA1 channel is to employ knockout models that specifically lack this channel. A TRPA1 knockout mouse does exist, where the exons required for proper function of TRPA1 were deleted [45]. The results up to now have all reported experiments conducted on rats. We therefore repeated the electrophysiology and Ca^2+^ imaging in the TRPA1-KO as well as the control wild-type C57Bl/6 J mice.

**Figure 4.**
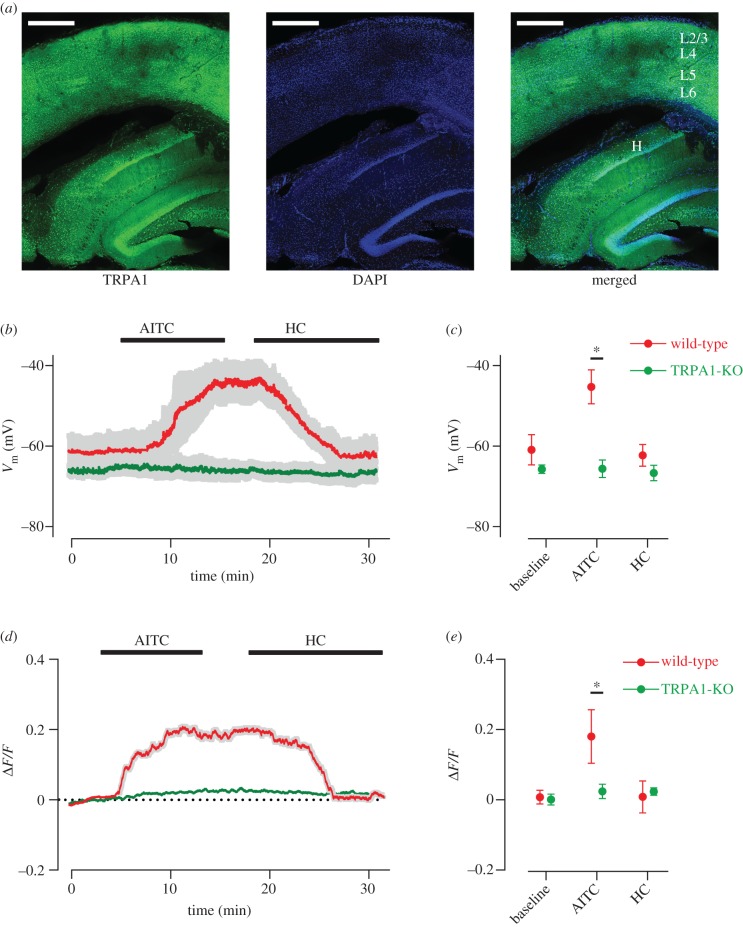
Expression of TRPA1 in mouse cortex and lack of functionality in the knockout. (*a*) Confocal images of a cortical slice (coronal section) from wild-type mouse capture anti-TRPA1 antibody (green) merged with nuclear staining with DAPI (blue). Scale bar, 500 µm in all panels. (*b*) Whole-cell recording of L5 pyramidal neurons of wild-type mice (red) reveal depolarization with TRPA1 agonist AITC. The shaded area indicates s.e.m. of *V*_m_ across neurons (*n* = 3). By replacing AITC with specific TRPA1 antagonist HC-030031 (HC), the membrane potential returned to its baseline values (hyperpolarized). Similar recording from L5 pyramidal neurons (*n* = 3) of TRPA1-KO mice (green) did not show a significant modulation in response to AITC or HC. (*c*) The mean *V*_m_ values and s.e.m. at each critical time point: at baseline (baseline, 2.5 min); after application of agonist (AITC, 15 min); and after application of antagonist (HC, 27.5 min). *p < 0.001, *n* = 3 for each circle. (*d*) Cortical neurons were loaded with Cal-520. Five minutes after the onset of imaging, AITC was applied to the bath, followed by washing and then by application of HC-030031 (HC). The grey shaded area indicates s.e.m. of Δ*F*/*F* across neurons (*n* = 153 and 156 for wild-type and TRPA1-KO, respectively) recorded in six imaging experiments. (*e*) Δ*F*/*F* measured at three time points after the onset of imaging: 2.5 min (baseline), 15 min (AITC) and 27.5 min (HC). Δ*F*/*F* is measured over a 1 s duration for each cell and averaged across cells. Error bars indicate s.d. of Δ*F*/*F* across neurons. *p < 0.001.

The procedures for slice preparation, electrophysiology and imaging were similar to those used on rats. The electrophysiology and Ca^2+^ imaging demonstrated the lack of functional modulation by the agonist and antagonist in the TRPA1-KO while replicating the earlier findings in the wild-type mice (figure 4). *In vitro* whole-cell recording of L5 pyramidal cells revealed systematic modulations of *V*_m_ in the presence of AITC and HC-030031 in the wild-type (red data points in figure 4*b*,*c*) but not in the TRPA1-KO (green data points in figure 4*b*,*c*). The *V*_m_ modulations in wild-type were qualitatively similar to those observed earlier in rats. Figure 4*d* illustrates the relative changes in fluorescence (Δ*F*/*F*) after application of AITC and HC-030031. As observed earlier in rat cortex, for the wild-type mouse cortex introducing AITC in the bath solution increased Δ*F*/*F*, indicating a rise in neuronal [Ca^2+^]_c_. After replacing AITC with 10 µM HC-030031, the neuronal [Ca^2+^]_c_ returned to its baseline values (red traces in figure 4*d*,*e*). Compared with the wild-type, the modulation in the neuronal [Ca^2+^]_c_ was minimal in the TRPA1-KO and did not return to baseline after application of HC-030031. There was a significant difference in AITC modulation across the population of 153 cells imaged from the wild-type, and 156 cells imaged from the TRPA1-KO (paired *t*-test, *p* < 0.001).

### 3.5. Two-photon photoswitching of optovin-loaded neurons

Altogether, the immunostaining, electrophysiological and Ca^2+^ imaging results support the idea that L5 pyramidal neurons exhibit functional TRPA1 channels. However, our results so far do not exclude the possibility of indirect activation of neurons by gating TRPA1 channels present in the cortical tissue including in glial cells and blood vessels. In other words, the observed changes in *V*_m_ and [Ca^2+^]_c_ may not be mediated by direct activation of TRPA1 channels of the recorded neuron. To rule out the possibility of an indirect TRPA1 activation in the cortex, we need a method where activation can be restricted to a single neuron. Targeted light application to photoswitch optovin in single cells provides this possibility.

**Figure 5.**
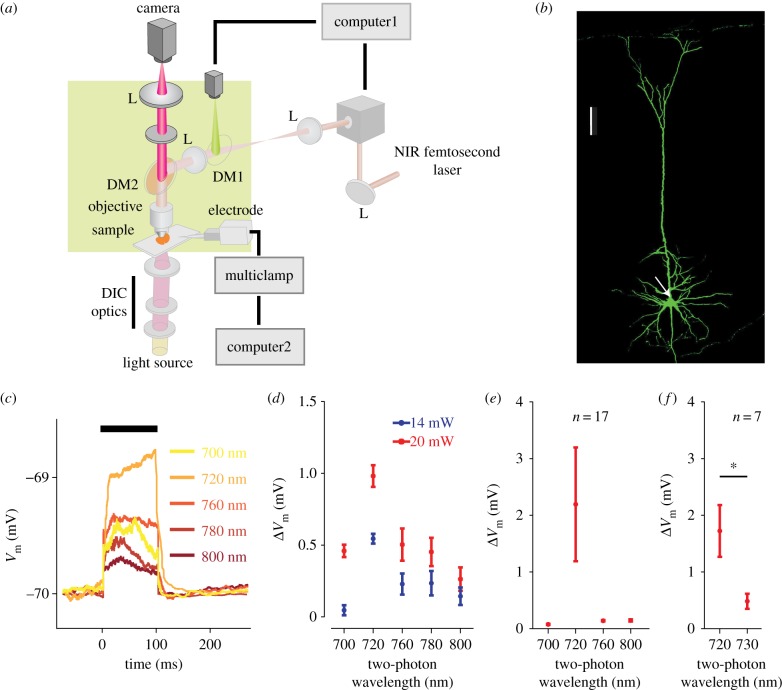
Two-photon photoswiching of optovin-loaded neurons. (*a*) Schematic of the custom-built two-photon set-up. After transferring the brain slice to the recording chamber, the L5 pyramidal neurons were visualized by applying light through differential interference contrast (DIC) optics using an Olympus BX50WI. This visualization is necessary for targeted whole-cell recording. The dichroic mirror 1 (DM1) reflects fluorescent light (*λ* < 650 nm) for two-photon imaging. DM2 passes near-infrared (NIR) light (*λ* > 900 nm) for DIC imaging and reflects the lower wavelengths (*λ* < 900 nm) for two-photon imaging and optovin photoswitching. The intensity and duration of NIR light is controlled by computer1 via a shutter. A separate computer (computer2) is used for acquisition and analysis of the electrophysiological data. L, lens. (*b*) Confocal images of a biocytin-loaded L5 pyramidal neuron (green). The arrow indicates the location where the NIR fs-pulsed laser was applied at approximately 1 µm diameter. Scale bar, 100 µm. (*c*) Two-photon activation of the optovin-loaded neuron shown in (*b*) at five excitation wavelengths. The neuron was loaded with 3 µM optovin. The two-photon excitation beam (20 mW) was applied from 0 to 100 ms (the thick horizontal line). Every line represents the mean *V*_m_ across 20 trials of light application. The depolarization was significantly higher at *λ* = 720 nm compared with all other wavelengths (all *p* < 0.001). (*d*) Changes in membrane potential (Δ*V*_m_) of the optovin-loaded neuron (from (*b*)) in response to two-photon photoswitching. The NIR fs-pulsed laser was applied at each wavelength/intensity combination for a block of 20 trials. These blocks were interleaved in a pseudorandom order and repeated three times to produce 60 trials per condition. Error bars indicate s.e.m. across trials (*n* = 60). The maximum depolarization was observed at *λ* = 720 nm (*p* < 0.001 compared with all other wavelengths). (*e*,*f*) As in (*d*) but across L5 pyramidal neurons. Error bars indicate s.e.m. of Δ*V*_m_ across neurons (*n* = 17, panel (*e*); *n* = 7, panel (*f*)) all loaded with 3 µM optovin. *p < 0.01.

Optovin is a photoactive molecule that gates TRPA1 after it is photoswitched by violet light [36]. The specificity for TRPA1 is supported by the finding that optovin photoswitching was significantly abolished in DRG neurons harvested from TRPA1 knockout mice [36]. Here, we replaced the violet light with an NIR fs-pulsed laser for a less scattered targeting. Localized photoswitching allows us to limit activation to TRPA1 channels in an optovin-loaded neuron (figure 5*a*). To achieve this, we performed *in vitro* whole-cell recording of L5 pyramidal neurons from rat somatosensory cortex using an internal solution containing optovin. We then applied the NIR fs-pulsed laser, 1 µm in diameter (see [40]), to the dendritic shaft of the patched neuron. The internal application of optovin along with the focused light ensures direct activation of TRPA1 channels restricted to the recorded neuron. The sample neuron shown in figure 5*b* was loaded with 3 µM optovin and excited at its dendritic shaft (the laser was focused at the location marked with the arrow). We found that 100 ms application of NIR fs-pulsed laser at wavelengths in the range *λ* = 700–800 nm modulated the neuron's membrane potential. Figure 5*c* shows the membrane potential averaged across 20 repetitions of laser stimulation at each wavelength. Minimal depolarization was induced at the longest wavelength (*λ* = 800 nm). The depolarization gradually increased with decreasing wavelength and peaked at *λ* = 720 nm. Further decrease in the wavelength below *λ* = 720 nm reduced the degree of depolarization. The depolarization also depended on the laser power (figure 5*d*): increasing the intensity of the two-photon excitation beam from 14 mW (blue) to 20 mW (red) produced a significant upward shift in the neuronal response. These findings were replicated across neurons: light activation was observed for all recorded L5 pyramidal neurons (*n* = 17) with the maximum neuronal response detected consistently at *λ* = 720 nm (figure 5*e*). As a control experiment, we applied the same protocol in the absence of optovin and found no effects of laser stimulation (see below). In the next step, we further investigate the optimal parameters for optovin photoswitching.

### 3.6. Optimal parameters for light activation of optovin

**Figure 6.**
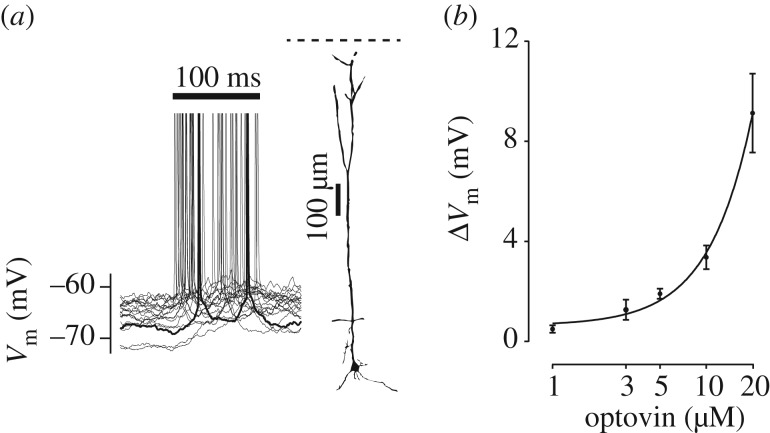
Dose dependency of optovin photoswiching. (*a*) A sample L5 pyramidal neuron loaded with 20 µM optovin. Laser application (*λ* = 720 nm, 20 mW) produced firing of action potentials across all trials. Action potentials are truncated and one of the traces is highlighted in bold. The recorded neuron is histologically reconstructed as L5 pyramidal. The horizontal dashed line indicates cortical surface. (*b*) The dose–response curve at different optovin concentrations. The curve shows the exponential fit to the data; note the logarithmic scale for optovin concentration. Error bars indicate s.e.m. of Δ*V*_m_ across neurons (*n* = 5 at each concentration).

We first determined how light activation depended on the cellular concentration of optovin. By increasing the concentration of optovin, Δ*V*_m_ increased significantly and even resulted in firing action potentials in some of the recorded cells. Figure 6*a* illustrates the firing profile of an example L5 pyramidal neuron along with its morphological reconstruction; this neuron was loaded with 20 µM optovin. Here, two-photon photoswitching with NIR fs-pulsed laser (*λ* = 720 nm, 20 mW, for 100 ms) produced reliable firing of action potentials across all trials. For this neuron, the membrane potential before and after each stimulation block remained stable at approximately −70 mV. There was however high trial-to-trial variability in the *V*_m_ during the stimulation block (figure 6*a*). To quantify how the excitation depended on the presence of optovin and its concentration, neurons were loaded with 0, 1, 3, 5, 10 and 20 µM optovin. As optovin is introduced through the internal pipette solution, only a single concentration could be applied to each neuron. A total of 30 neurons were recorded (*n* = 5 for each concentration). Changes in membrane potential were measured after removing any action potentials. Figure 6*b* illustrates how the degree of light-induced depolarization increased with optovin concentration. The dose–response curve is plotted on a logarithmic axis and does not show the recordings that were performed in the absence of optovin; in the absence of optovin laser stimulation at *λ* = 720 nm wavelength and with maximum light power (20 mW) did not produce any depolarization in the recorded neurons (*n* = 5; before and after light paired *t*-test *p* > 0.2).

### 3.7. Optovin photoswitching activates neurons through TRPA1 channels

**Figure 7.**
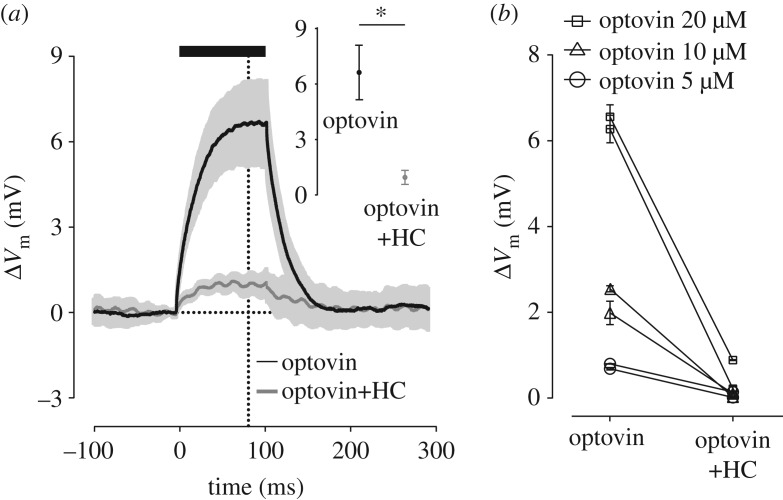
Optovin photoswitching activates neurons through TRPA1 channels. (*a*) An optovin-loaded neuron is depolarized with light (black). Optovin concentration was 20 µM. Light application was from 0 to 100 ms as indicated by the thick line. At the end of 20 trials of light application, HC-030031 (HC, 10 µM) was introduced to the bath and after 10 min of exposure the light stimulation protocol was repeated. Neuronal depolarization to light diminished in the presence of HC-030031 (grey). The inset shows Δ*V*_m_ measured 20 ms before the light offset. The shaded area and the error bars (inset) indicate s.e.m. of Δ*V*_m_ across trials (*n* = 60). *p < 0.001. (*b*) The effect of HC-030031 is reproduced across neurons (*n* = 6) at three different concentrations of optovin.

Finally, to confirm that optovin activated these neurons via its specific interaction with TRPA1, we repeated the whole-cell recordings in the presence or absence of the selective TRPA1 blocker HC-030031 (10 µM). The neuronal response was significantly reduced in the presence of the blocker (figure 7*a*, paired *t*-test *p* < 0.0001) and this was true for all recorded neurons loaded at three different concentrations of optovin (figure 7*b*, *n* = 6, all *p*-values < 0.02).

Altogether, these experiments indicate the expression of TRPA1 in rodent somatosensory cortex and demonstrate that optovin can be used to optically control the activity of pyramidal neurons at high spatial and temporal resolution. The two-photon photoswitching of optovin thus provides a precise method for investigating the physiological and pathological roles of TRPA1 in the mammalian cortex.

## 4. Discussion

To understand a neuronal circuit, a productive approach is to perturb the activity of a specific element, i.e. a dendrite of a neuron or a subpopulation of cells within the circuit, and to quantify the functional consequence of this perturbation. Photostimulation techniques allow perturbation of activity at high spatio-temporal resolution; light has been used to (i) locally uncage neurotransmitters [46], (ii) open photosensitive membrane channels (optogenetics) [47] and (iii) activate synthetic photoswitching molecules which in turn affect endogenous channels [48]. Here, we used optovin photoswitching for selective targeting of TRPA1 channels that were endogenous to the cortical neurons.

TRPA1 was previously shown to be highly expressed in large cortical vessels and to produce arterial dilatation when activated [41]. Our immunostaining confirmed the prominent presence of TRPA1 in the cortical vasculature. We found that TRPA1 was also expressed in the neurons across layers of the rodent somatosensory cortex, and thus examined its function by application of TRPA1 agonist, AITC, and antagonist, HC-030031. Both two-photon Ca^2+^ imaging and whole-cell electrophysiology revealed TRPA1 modulation of neuronal activity: application of the AITC increased intracellular Ca^2+^ concentration and depolarized the cell membrane, and these effects were blocked by HC-030031, both in the rat cortex and that of wild-type mouse. Importantly, these modulations were absent in the TRPA1-KO mouse. Finally, by targeting TRPA1 through optovin photoswitching, we achieved localized and temporally precise modulation of activity in individual L5 pyramidal neurons. Our results thus extend a previous optovin activation of TRPA1 in the zebrafish [36] to rodents and provide a method to further investigate the role of TRPA1 in the sensory cortex. Previously, violet light, with a wavelength of *λ* = 385–405 nm, was used for single-photon photoswitching of optovin. Light absorption and scattering by the tissue is higher at wavelengths in the visible range [49]. To allow sufficient light penetration in a highly scattering tissue such as the mammalian cortex, an increase in intensity is necessary which can result in cortical damage [50]. We applied an NIR fs-pulsed laser for two-photon excitation at *λ* = 720 nm. NIR light has reduced scattering and absorption through cortical tissue resulting in better penetration and a more localized excitation.

The presence of TRPA1 is well established in the peripheral nervous system and a wide range of non-neuronal tissues [7–15]. TRPA1 promotes the process of neurogenic inflammation and enhances the acute or persistent pain sensation [1]. TRPA1 is also thought to be involved in noxious cold sensation [16,51]. TRPA1 is expressed in the inner hair cells [16]. However, despite earlier evidence for its contribution to hearing [16], its functional role is unclear due to the observation of normal hearing in TRPA1-KO mice [45]. In the central nervous system, whole-brain PCR has confirmed the presence of TRPA1 protein but its expression and function has been investigated mostly in the hippocampus and the brain stem [7,14,23]. In hippocampal neurons, TRPA1 is involved in cannabiod receptor activation [7]. TRPA1 is linked to GABA transport in hippocampal asterocytes, and its activation increases the extracellular GABA concentration, which in turn lowers the effciency of inhibitory synapses among interneurons [14]. In supraoptic nucleus, TRPA1 agonists produce potentiation of excitatory synaptic inputs in the magnocellular neurosecretory cells possibly through modulation of glutamate release [23]. In the brain stem, TRPA1 is expressed in the capsaicin-sensitive and capsaicin-insensitive neurons of the nucleus tractus solitarius and is involved in the release of glutamate from these neurons [52]. The common observation across all these studies is an enhanced level of excitability in the circuit by TRPA1 activation.

TRPA1 is widely investigated and better understood in non-mammalian spices, where it is associated with several sensations and a range of behavioural responses [7]. In *Caenorhabditis* *elegans*, TRPA1 is known to mediate the nematode's behavioural response to mechanosensory stimuli [53]. In *Drosophila* (fruit flies), TRPA1 contributes to multiple sensations including nociception, olfaction, noxious cold sensation, negative geotaxis (movement against gravity) and cardiac mechanosensation [54–59]. In snakes, TRPA1 is expressed in the pit organ which is responsible for infrared detection [60].

Beyond its known physiological roles, TRPA1 is implicated in a number of pathological conditions including the generation of asthma, joint oedema, osteoarthritis and skin inflammatory diseases such as acute inflammatory response and allergic contact dermatitis [16–18,37,41,44,61–64]. Similar to its involvement in other tissues and organs, cortical TRPA1 may contribute to physiological functions such as signal processing in sensory cortex and motor behaviours or in developing pathologies such as in Alzheimer's disease [65], brain oedema or oxidative-stress induced neuronal damage.

The recent structural discovery of TRPA1 with electron cryomicroscopy revealed a TRP-domain helix (the transmembrane domain) similar to that of TRPV1 [26]. As well as possessing structural similarities, TRPA1 and TRPV1 are co-expressed in many neuronal and non-neuronal sites [66]. In fact TRPV1 is present in 97% of TRPA1 expressing sensory neurons [51]. It is therefore not surprising that these two channels are involved in similar functions [26,67]. In the peripheral nervous system, both channels contribute to the processing of noxious stimuli including mechanical, thermal and chemical sensations, as well as in pain and neurogenic inflammation [66]. TRPA1 and TRPV1 are also co-expressed in a number of non-neuronal cell types including keratinocytes, endothelial cells and the smooth muscle cells in vessels and have again been implicated in similar pathologies such as joint inflammation and oedema [66]. TRPV1 is expressed in cortex, hippocampus, cerebellum, mesencephalon, hindbrain and olfactory bulb [68]. In the hippocampus and brain stem, TRPA1 and TRPV1 both enhance synaptic glutamate release and are linked to the attenuation of cannabinoid receptors [7,23,52,69,70]. In the human cortex and that of other mammals, TRPV1 is known to be involved in synaptic transmission [70–72]. It is likely that TRPA1 exhibits shared functionality with TRPV1 also in the cortex.

The diverse range of neuronal and non-neuronal cell types that express TRPA1 [66] indicates a highly diverse functional role for this channel. Our findings demonstrate the expression and the functional activation of TRPA1 in rodent cortex. However, these results do not necessarily demonstrate a direct role for this channel in cortical functions such as a contribution to sensory processing or behaviour. It is possible that TRPA1 is activated during pathological conditions such as oedema and brain trauma. This would be consistent with the broad role of TRPA1 in inflammation, infection and immunity observed across a range of tissues. It remains to be investigated how TRPA1 channels interact with other channels in the brain in order to contribute to cortical function and to pathological conditions. To address these questions future experiments could utilize optovin photoswitching in cortical neurons *in vivo* to specifically target TRPA1 channels at high spatio-temporal precision.

## Data accessibility

Datasets supporting this article can be found at the central data repository of the Australian National University, see http://dx.doi.org/10.4225/13/58e1c70673270.
